# Genome-wide identification and expression patterns of the laccase gene family in response to kiwifruit bacterial canker infection

**DOI:** 10.1186/s12870-023-04606-z

**Published:** 2023-11-27

**Authors:** Zhuzhu Zhang, Youhua Long, Xianhui Yin, Weizhen Wang, Wenzhi Li, Lingli Jiang, Xuetang Chen, Bince Wang, Jiling Ma

**Affiliations:** 1 Research Center for Engineering Technology of Kiwifruit, Institute of Crop Protection, College of Agriculture, Guizhou University, Guiyang, 550025 China; 2 Teaching Experiment Farm, Guizhou University, Guiyang, 550025 China

**Keywords:** Kiwifruit canker, Laccase, Gene family, *Pseudomonas syringae* pv. *actinidiae*, Gene expression

## Abstract

**Background:**

Kiwifruit bacterial canker, caused by *Pseudomonas syringae* pv*. actinidiae* (Psa), is a destructive disease worldwide. Resistance genes that respond to Psa infection urgently need to be identified for controlling this disease. Laccase is mainly involved in the synthesis of lignin in the plant cell wall and plays a prominent role in plant growth and resistance to pathogen infection. However, the role of laccase in kiwifruit has not been reported, and whether laccase is pivotal in the response to Psa infection remains unclear.

**Results:**

We conducted a bioinformatics analysis to identify 55 laccase genes (*AcLAC1–AcLAC55*) in the kiwifruit genome. These genes were classified into five cluster groups (I–V) based on phylogenetic analysis, with cluster groups I and II having the highest number of members. Analysis of the exon–intron structure revealed that the number of exons varied from 1 to 8, with an average of 5 introns. Our evolutionary analysis indicated that fragment duplication played a key role in the expansion of kiwifruit laccase genes. Furthermore, evolutionary pressure analysis suggested that *AcLAC* genes were under purifying selection. We also performed a *cis*-acting element analysis and found that *AcLAC* genes contained multiple hormone (337) and stress signal (36) elements in their promoter regions. Additionally, we investigated the expression pattern of laccase genes in kiwifruit stems and leaves infected with Psa. Our findings revealed that laccase gene expression levels in the stems were higher than those in the leaves 5 days after inoculation with Psa. Notably, *AcLAC2*, *AcLAC4*, *AcLAC17*, *AcLAC18*, *AcLAC26*, and *AcLAC42* showed significantly higher expression levels (*p* < 0.001) compared to the non-inoculated control (0 d), suggesting their potential role in resisting Psa infection. Moreover, our prediction indicated that 21 kiwifruit laccase genes are regulated by miRNA397, they could potentially act as negative regulators of lignin biosynthesis.

**Conclusions:**

These results are valuable for further analysis of the resistance function and molecular mechanism of laccases in kiwifruit.

**Supplementary Information:**

The online version contains supplementary material available at 10.1186/s12870-023-04606-z.

## Background

Kiwifruit (*Actinidia chinensis*) has become a popular fruit among consumers given its unique nutritional value. With increasing demand and sales, it is becoming a fruit crop of immense value [1]. Presently, China ranks first worldwide in kiwifruit planting area and yield, making it a prominent player in the rapidly expanding kiwifruit industry [2]. However, with the expansion of the kiwifruit cultivation area and the continuous increase in yield, diseases in kiwifruit orchards are becoming widespread, especially kiwifruit bacterial canker, which is particularly devastating [3]. In the major kiwifruit cultivation areas of New Zealand [4], Italy [5], Korea [6], and other countries [7, 8] and in Shaanxi [9], Sichuan [10], Guizhou [11], and other regions of China, kiwifruit canker disease tends to be severe. Kiwifruit bacterial canker is caused by *Pseudomonas syringae* pv*. actinidiae* (Psa), which is highly pathogenic, remains hidden, and spreads rapidly, rendering enormous economic burden on the kiwifruit industry.

After the exploration of various control methods, researchers have pointed out that one of the most effective countermeasures against Psa infection is the selection of disease-resistant varieties of kiwifruit [12, 13]. However, there are few reports on kiwifruit disease resistance-related genes. Therefore, it is imperative to explore and identify resistance-related genes to establish a solid foundation to understand the resistance mechanisms in kiwifruit.

Lignin is the primary phenolic polymer comprising the secondary cell wall of vascular plants [14]. It impedes pathogenic bacteria from degrading the cell wall and effectively obstructs material exchange between pathogens and host plants, thereby contributing to plant disease resistance [15, 16]. Laccase (E.C. 1.10.3.2) is the key enzyme in lignin synthesis and belongs to the copper blue oxidase family [17, 18]. Extensive research has been conducted on the defense-related functions of many laccases in various crops. For example, 17 laccase genes are found in *Arabidopsis thaliana*, of which *AtLAC4*, *AtLAC11*, and *AtLAC17* are responsible for lignin polymerization [19], and 30 laccase genes are found in rice, of which *OsLAC10* synthesizes lignin and participates in abiotic stress response [20]. A total of 95 laccase genes have been identified in wheat, of which 14 have a positive response during *Fusarium graminearum* infection, reducing damage to plant cell walls [21]. However, there are no reports of laccase genes in kiwifruit. Moreover, the availability of the high-quality whole-genome sequence of kiwifruit [22, 23] allows for a comprehensive analysis of kiwifruit laccase genes.

Therefore, the purpose of this study was to identify members of the laccase gene family in the kiwifruit genome and further investigate the resistance-related functions and regulatory mechanisms of laccases in kiwifruit disease resistance. The phylogenetic relationship, structural characteristics, chromosome location, *cis*-elements, and expression pattern of kiwifruit laccase genes under Psa infection were analyzed to identify candidate genes for kiwifruit bacterial canker resistance. The study results provide a theoretical basis for revealing the function of kiwifruit laccases, potentially contributing to the breeding of disease-resistant kiwifruit varieties.

## Results

### Psa Infection and degradation of kiwifruit stem lignin

**Fig. 1:**
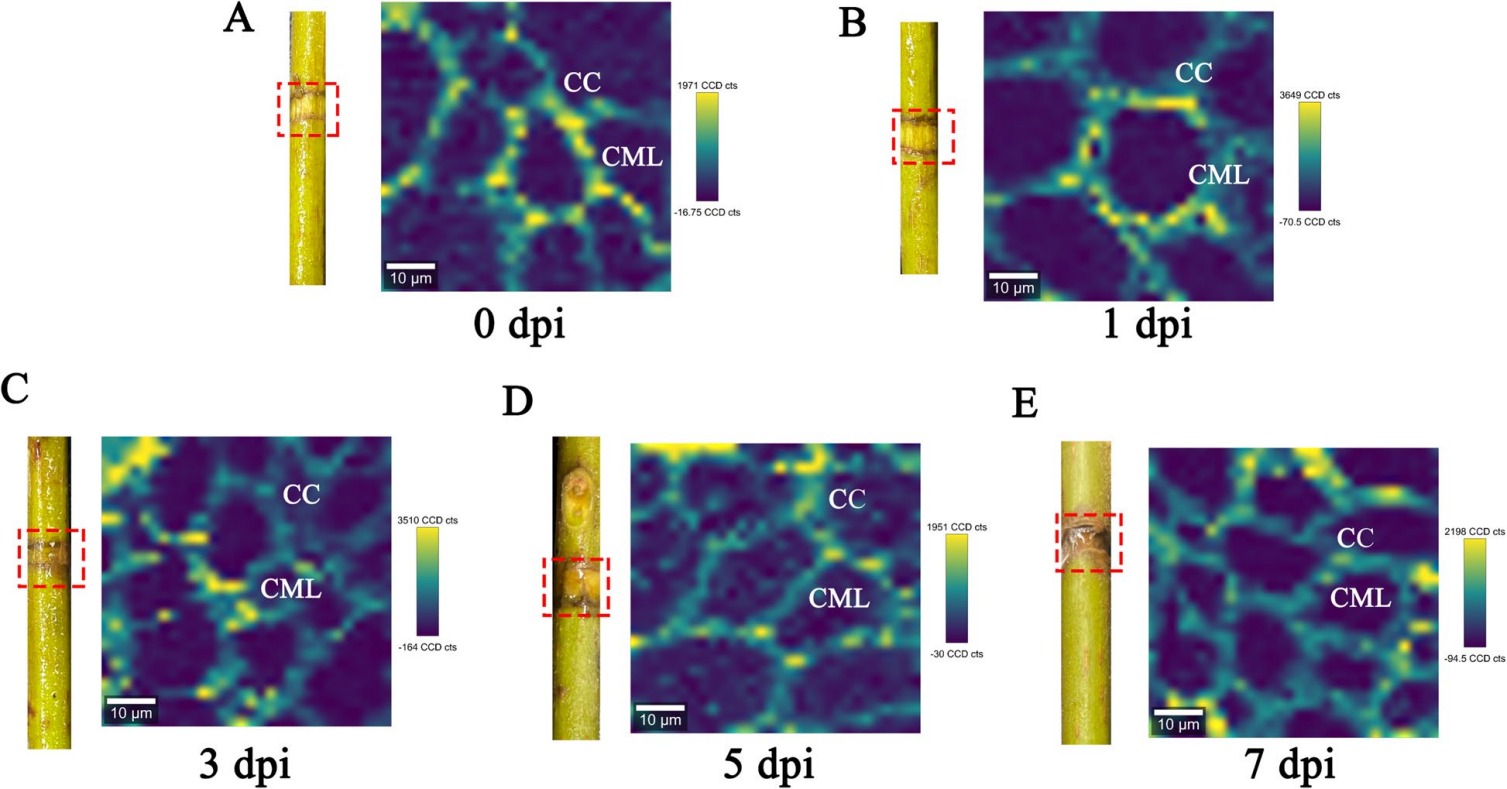
Raman imaging of Psa-infected kiwifruit stems. CC: cell corner; CML: cell intercellular layer

**Fig. 2:**
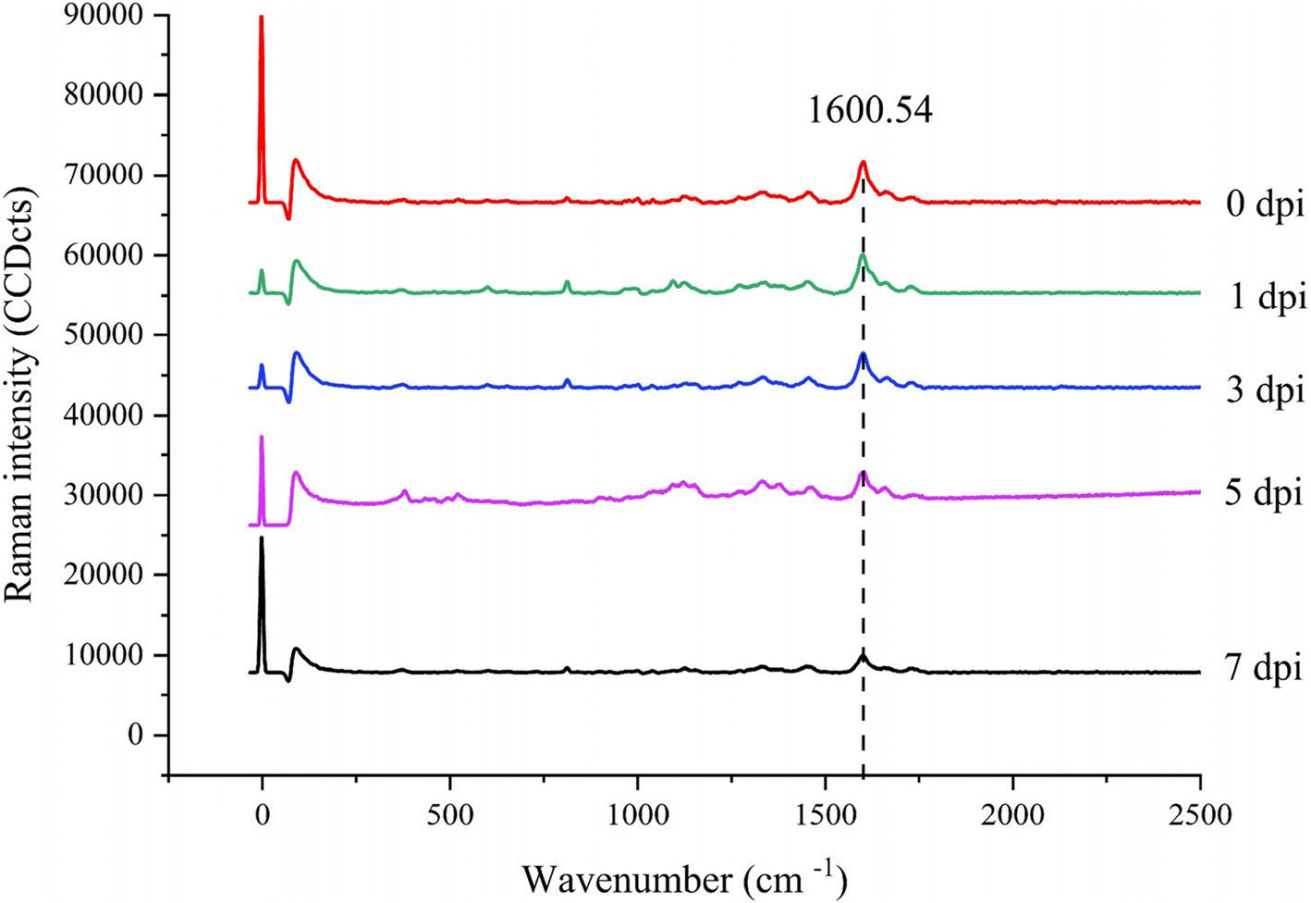
Raman spectra of *Pseudomonas syringae* pv*. actinidiae* (Psa)-infected kiwifruit stems

**Fig. 3:**
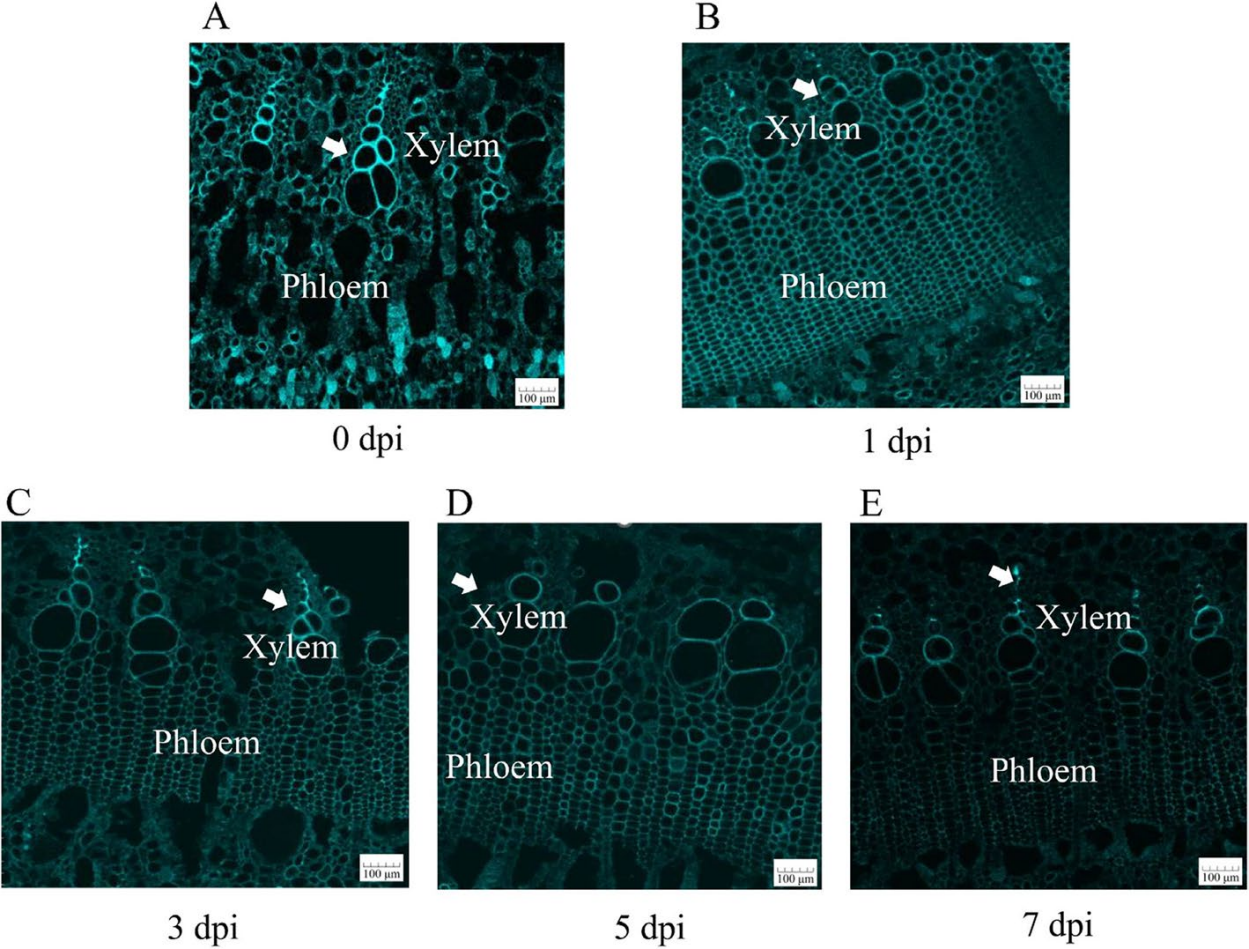
Lignin autofluorescence

Confocal Raman microscopy was used to observe changes in stem lignin after Psa infection by detecting the stretching vibration wavelength of the lignin aromatic ring near 1600 cm^−1^ [24], and images were acquired. As can be seen from the infection phenotype (Fig. 1), barely any difference in symptoms was observed 1 day after infection, like that in the control. With the increase in infection days, the lesion area gradually expanded, with browning on day 3 of inoculation. Some milky white bacterial pus appeared on days 5 and 7. However, the internal structure and lignin content of infected kiwifruit stems instantly changed to a considerable extent. These changes were reflected in Raman signals and imaging. With the increase in infection days, the Raman signal intensity gradually decreased. After 7 days of infection, the lignin signal intensity reached the lowest value, and the lignin content was also at its lowest (Fig. 2). Raman imaging visually displays the distribution and content of lignin in the cell wall at the cell level, and the more yellow the cell area, the higher the content of lignin. Raman imaging showed that the structure of the cell wall of uninfected kiwifruit stems was complete, the outline was clear, and there was high lignin distribution in the cell corner and the intercellular layer. After 5 days of infection, the intercellular layer of lignin disappeared and only existed in the corners of cells. After 7 days of infection, lignin disappeared in the corner and cell wall areas, indicating that it was almost completely degraded (Fig. 1). These results indicated that Psa infection of kiwifruit stem may mainly degrade cell wall lignin, and the degradation sequence is from the intercellular layer to the cell corner. As shown in Fig. 3, the fluorescence intensity of the xylem and phloem was brighter, indicating a higher lignin content. However, due to technical problems with the sectioning, the xylem cells in the figure are not completely visible. Nonetheless, the overall spontaneous fluorescence intensity was high (Fig. 3A). The fluorescence intensity of the phloem and xylem decreased as the duration of Psa infection increased. By the 7th day of infection, some fluorescence remained in the phloem, but the fluorescence intensity in the xylem region increased. This suggested that the lignin content in the xylem was initially affected by Psa infection, followed by that in the phloem.

### Identification of laccase genes and their physicochemical properties

Fifty-five laccase gene family members were identified in the kiwifruit genome and were named *AcLAC01*–*AcLAC55* (Supporting Information, Table S1) according to their chromosomal position. The lengths of the amino acid sequences of the corresponding proteins were between 454 and 598 amino acids, and the isoelectric points and molecular weights were 4.71–9.78 and 50.04–69.71 kDa, respectively. Subcellular localization prediction results showed that 39 laccase genes were located extracellularly and corresponded to secreted proteins, and 16 genes were located on the plasma membrane. Except for *AcLAC06*, *AcLAC31*, and *AcLAC50*, the remaining genes appear to encode signal peptides. The laccase genes of kiwifruit encode mostly hydrophilic amino acids.

### Phylogenetic and gene structure analyses

**Fig. 4:**
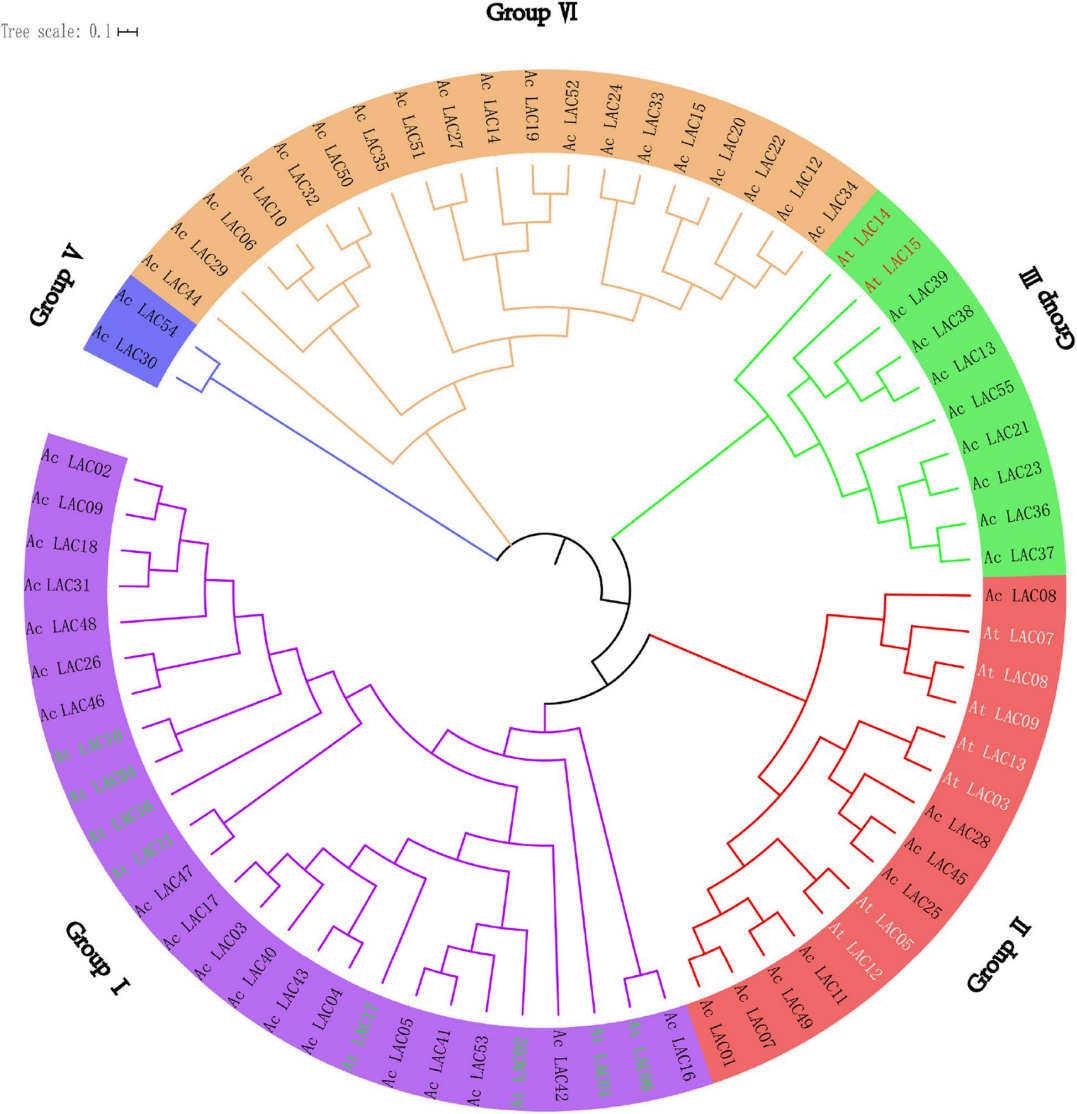
Phylogenic tree of laccase family members in *Arabidopsis thaliana* and *Actinidia chinensis*. At: *Arabidopsis thaliana*; Ac: *Actinidia chinensis*

**Fig. 5:**
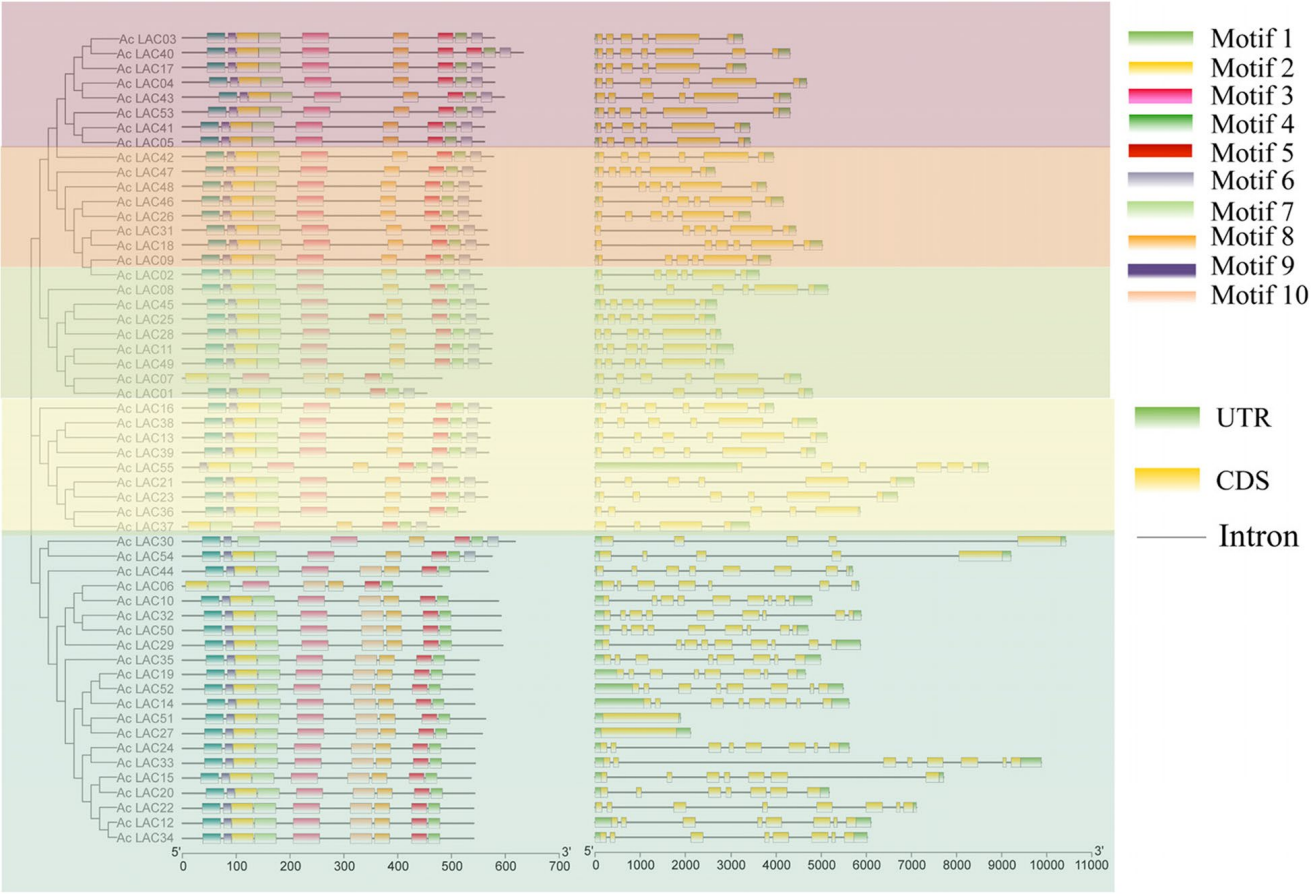
Gene structure analysis of kiwifruit laccase genes

Using MEGAX software, the sequences of 55 kiwifruit and 17 * Arabidopsis thaliana* laccase gene family members were aligned, and phylogenetic trees were constructed using neighbor-joining and EvolView. These genes could be divided into five groups, consisting of 26, 15, 10, 19, and 2 genes (Fig. 4). The gene structures of 55 laccase genes in kiwifruit were analyzed. The number of exons found ranged from one to eight, with most having six, and they had a common conserved domain (Fig. 5).

### Three-dimensional model analysis of laccase

**Fig. 6:**
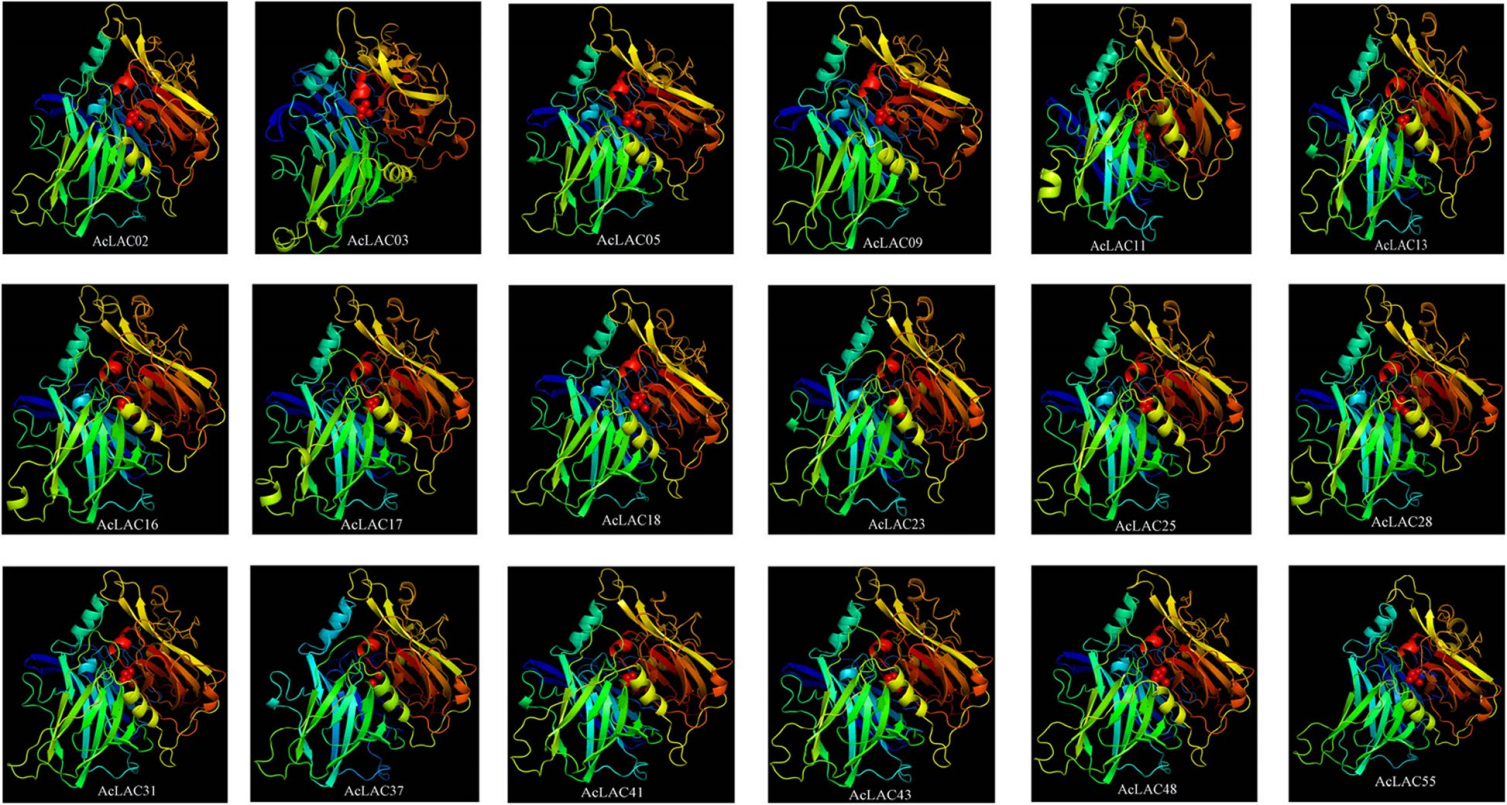
Three-dimensional (3D) structure modeling of kiwifruit laccase proteins. Alpha helix: Spring shape, Beta Strand: Arrow shape

Three-dimensional (3D) structural homologous modeling of the amino acid sequences of kiwifruit laccases showed a high similarity of amino acid-sequence tertiary structure. Therefore, representative sequences were selected (Fig. 6). Each of their sequences had three α-helices and at least 20 β-folds, which were very similar in 3D structure. Therefore, we speculate that the function of laccases in kiwifruit may be redundant or similar.

### Chromosome location

**Fig. 7:**
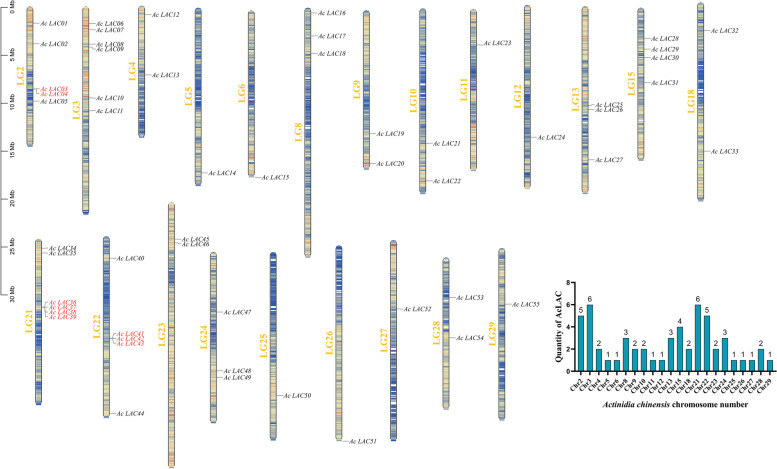
Chromosomal distribution of laccase genes

Laccase genes are unevenly and irregularly distributed on kiwifruit chromosomes. In addition to chromosomes 1, 7, 14, 16, 17, 19, and 20, other chromosomes also contain members. Chromosomes 3 and 21 contain six members each, and chromosomes 2 and 22 contain five members each. This indicates that the number of laccase genes on each chromosome is independent of chromosome size (Fig. 7).

### Collinearity and evolution analyses

**Fig. 8:**
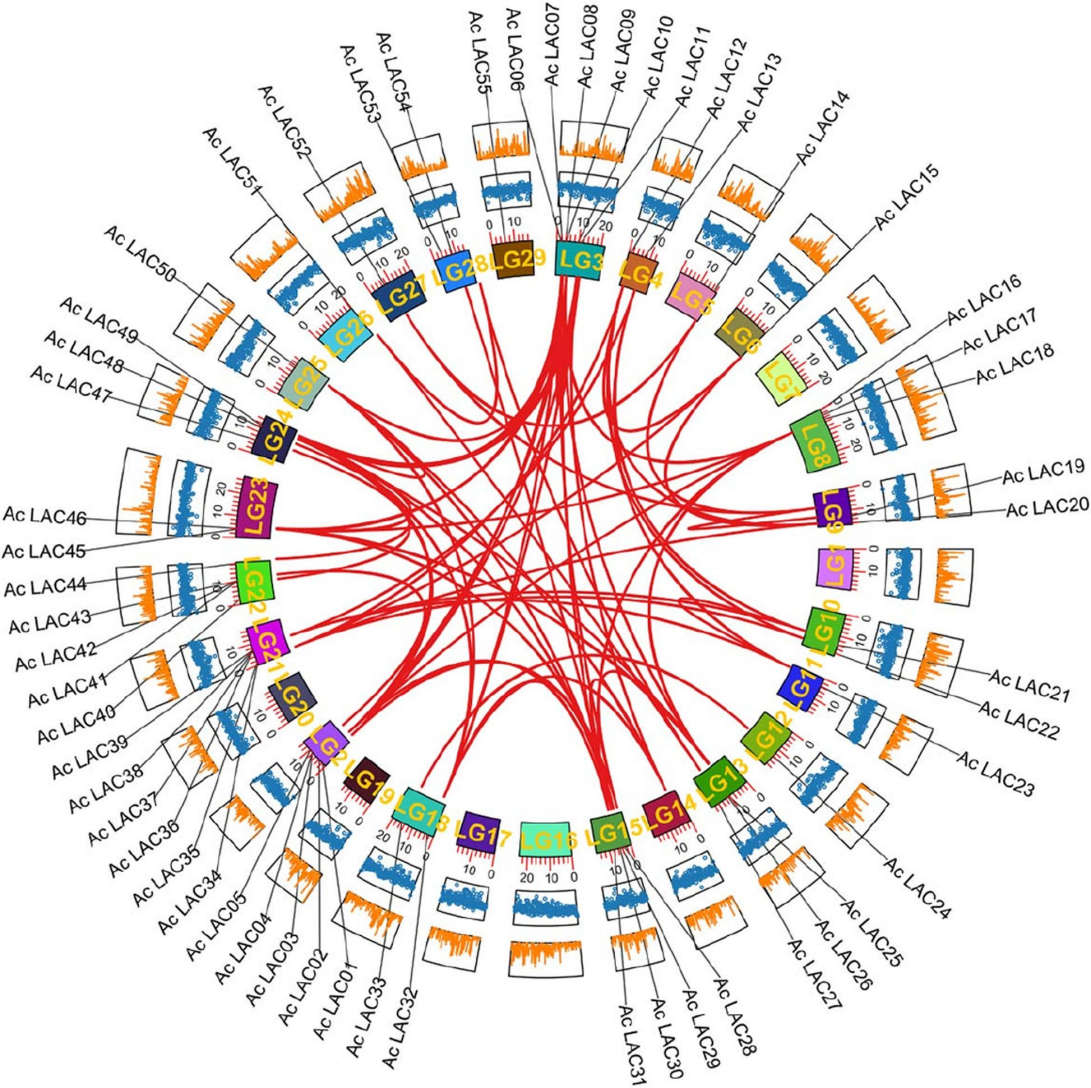
Collinearity of laccase gene pairs. Note: From inside out: chromosomes, gene density, GC content

**Fig. 9:**
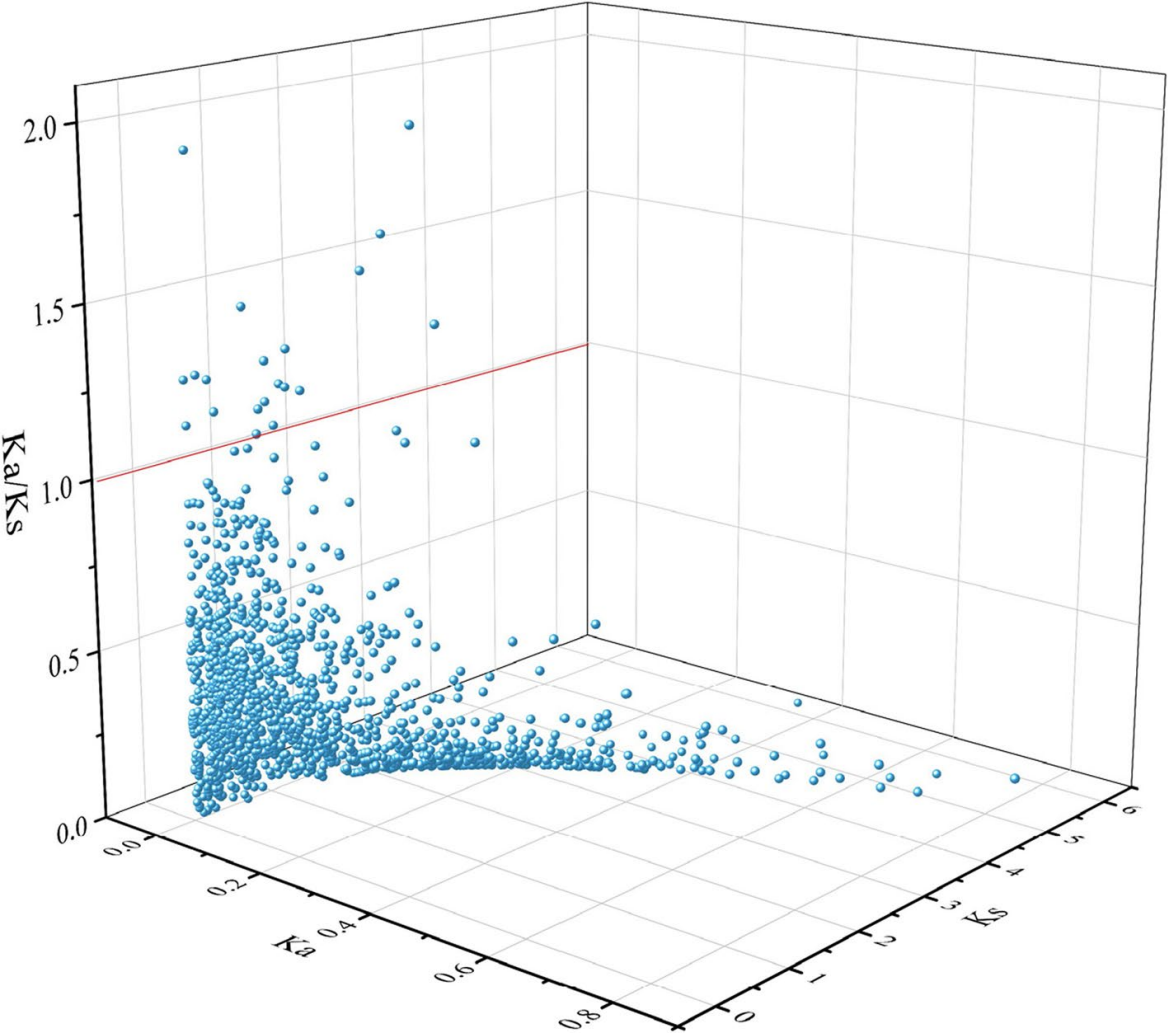
Selection pressure analysis of the laccase gene family in kiwifruit

**Fig. 10:**
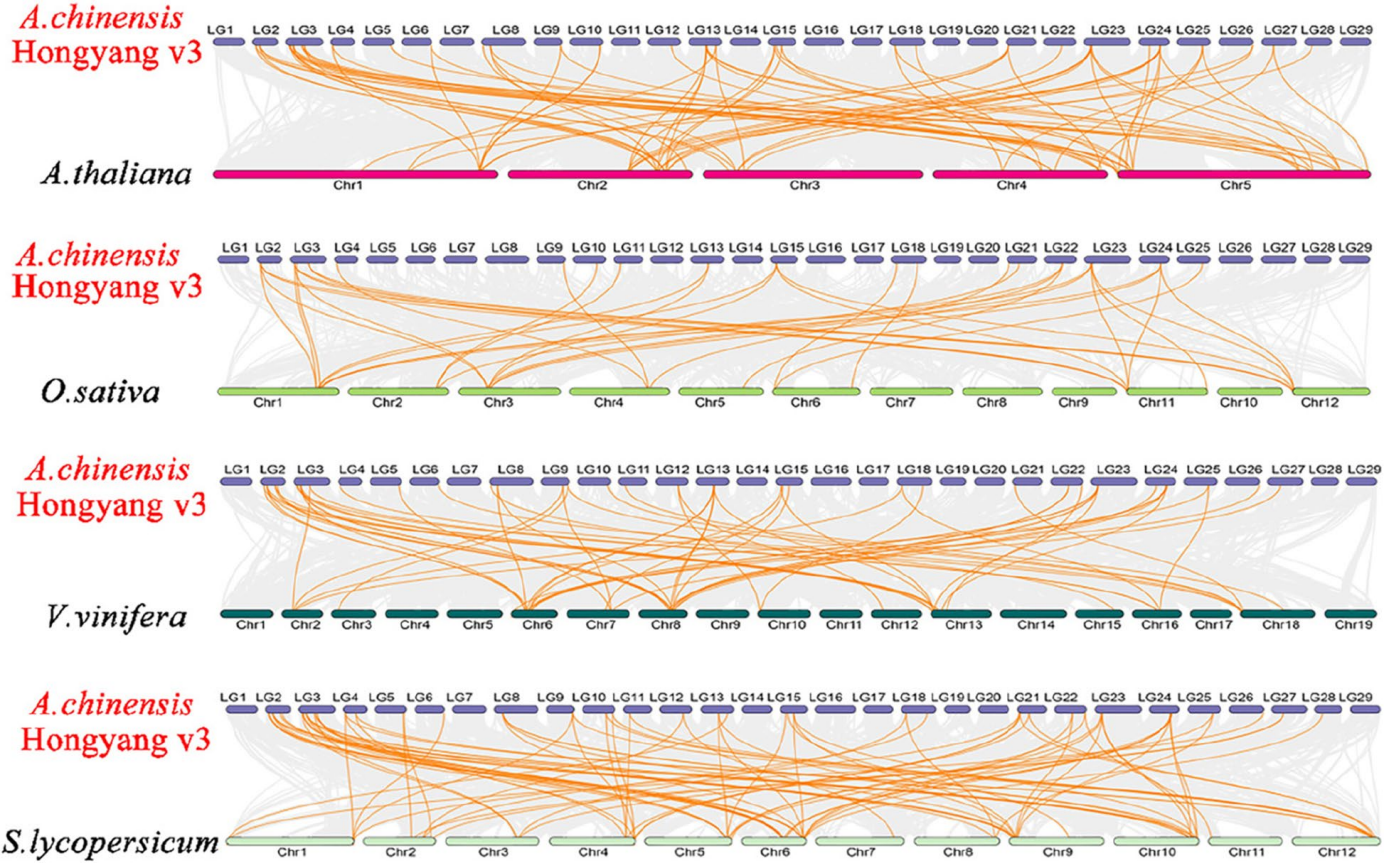
Laccase gene homology analysis of kiwifruit and four representative plants. Gray lines in the background indicate collinear blocks in kiwifruit and other plant genomes, while orange lines highlight syntenic laccase gene pairs. Species names prefixed with *A.thaliana*, *O.sativa*, *V.vinifera*, and *S.lycopersicum* denote *Arabidopsis thaliana*, *Oryza sativa*, *Vitis vinifera*, and *Solanum lycopersicum*, respectively

The MCScanX method was used to analyze the gene duplication events of kiwifruit, and 74 gene duplication events were identified. We also identified 68 repeat gene pairs and six tandem repeats (Fig. 8). These results suggest that gene fragment replication events are a major driver of laccase gene evolution in kiwifruit. Furthermore, most homologous laccase genes are paired with Ka/Ks < 1 (Fig. 9), indicating that the laccase gene pair may have undergone purification selection during evolution, which is crucial in maintaining the conserved structure. To further explore the phylogenetic mechanism of laccase gene replication in kiwifruit, we constructed a comparative map of the homology of four representative species (Fig. 10), including one monocot (*Oryza sativa* L*.*) and three dicots (*Arabidopsis thaliana*, tomato [*Solanum lycopersicum*], and grape [*Vitis vinifera* L.]) (See Supporting Table S2). Among these species, 92 laccase genes in tomato had a collinear relationship with kiwifruit, followed by *Arabidopsis thaliana* (77), grape (60), and rice (36). Compared with monocotyledonous rice, kiwifruit, a dicotyledonous plant, has a higher number of genes collinear with the other three dicotyledonous plants than with the monocotyledonous plant, indicating that collinear gene pairs existed before the evolutionary separation of monocotyledonous and dicotyledonous plants.

### Prediction of the *cis*-acting elements of laccase genes in kiwifruit

**Fig. 11:**
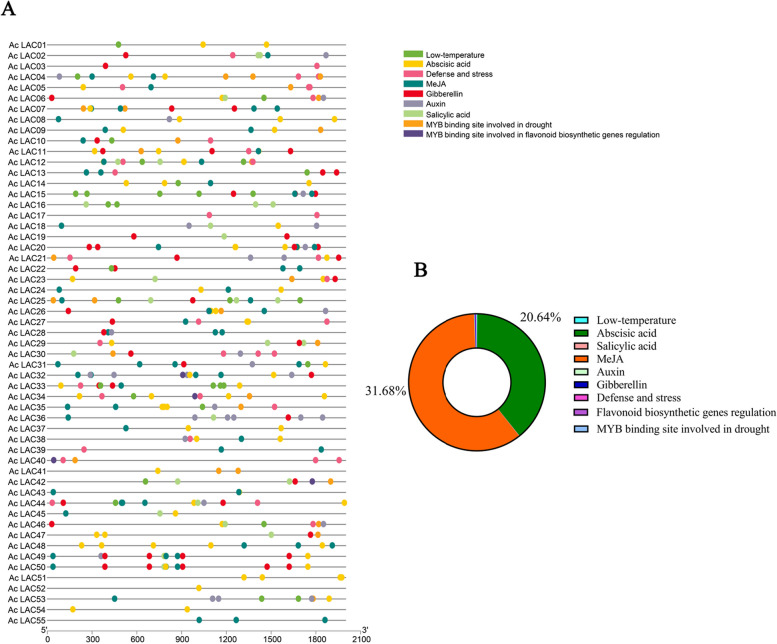
Analysis of *cis*-acting elements in the putative laccase gene promoter*.* **A**: Distribution of *cis*-acting elements in the promoters of the kiwifruit laccase gene family. **B**: Pie chart of *cis*-acting elements in the promoters of the kiwifruit laccase gene family

This study identified nine *cis*-acting elements of genes responding to biotic and abiotic stress, growth and development, and plant hormone responses (Fig. 11). Several elements associated with plant hormone signaling pathways were predicted, such as methyl jasmonate (MeJA), abscisic acid (ABA), salicylic acid (SA), gibberellin (GA), and auxin (IAA). There were 138 MeJA response elements (31.68%), 90 ABA response elements (20.64%), 50 GA response elements, 33 auxin response elements, 36 defense stress response elements, and a few flavonoid contract pathway response elements. This implies that most laccases can participate in MeJA and ABA-mediated signaling pathways and that laccases in kiwifruit are associated with various plant stress responses. It is also predicted that some elements can participate in various abiotic stresses (drought and low temperature).

### Laccase genes with miRNA targets

miR397, miR408, and miR857 have been confirmed to target plant laccase genes [25]. Among them, miR397 is a negative regulator of lignin content [26, 27]. To understand the miR397-mediated post-transcriptional regulation of kiwifruit laccase genes, we searched all the targets of miR397 (Supporting Information, Table S3).

### Expression pattern of laccases genes under Psa infection

**Fig. 12:**
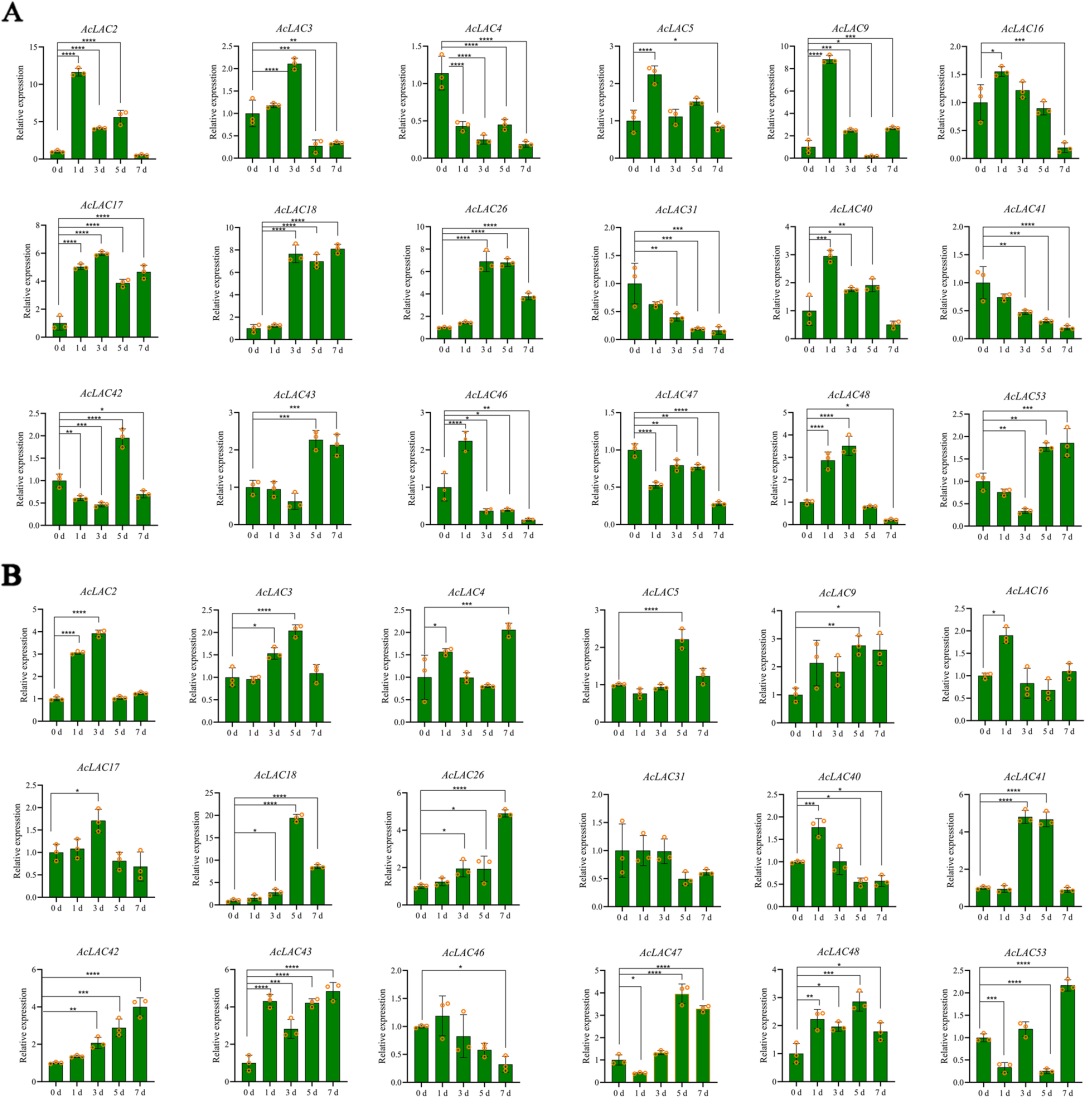
Expression of laccase in kiwifruit stems and leaves infected with Psa. The error bar represents the standard deviation of biological repetition (*n* = 3). *, * *, and * * * represent the statistical difference when *p* values are less than 0.05, 0.01, and 0.001, respectively. **A**: Relative gene expression of Psa-infected kiwifruit stems with incremental time. **B**: Relative gene expression of Psa-infected kiwifruit leaves with incremental time

To reveal the disease resistance function of laccases in kiwifruit bacterial canker, RT-qPCR was used to analyze the relative expression of laccase genes changes in kiwifruit leaves and stems after Psa infection (Fig. 12). In stem tissues (Fig. 12A), the expression levels of *AcLAC2, AcLAC3, AcLAC5, AcLAC9, AcLAC16, AcLAC17, AcLAC18, AcLAC26, AcLAC40, AcLAC46*, and *AcLAC*48 initially increased and then declined, with *AcLAC2*, *AcLAC5*, *AcLAC9*, *AcLAC40*, and *AcLAC46* reaching their peak expression (*p* < 0.001) after 1 day of Psa infection. Conversely, the expression levels of *AcLAC4*, *AcLAC42*, *AcLAC47*, and *AcLAC53* initially decreased and then increased, with *AcLAC42* and *AcLAC53* showing the lowest expression after 3 days of Psa infection. Notably, *AcLAC2*, *AcLAC4*, *AcLAC17*, *AcLAC18*, *AcLAC26*, and *AcLAC42* compared to the non-inoculated control (0 d), showed significantly higher expression levels (*p* < 0.001). In leaves of kiwifruit (Fig. 12B), the expression levels of *AcLAC5*, *AcLAC31*, *AcLAC41*, *AcLAC47*, and *AcLAC53* showed a decreasing trend followed by an increasing trend with the duration of infection, while the other genes exhibited an increasing trend followed by a decreasing trend. Additionally, the expression level of *AcLAC42* gradually increased with the duration of infection.

## Discussion

Plant laccase is a multifunctional oxidase, mainly involved in lignin synthesis [14, 28], flavonoid metabolism [29], and trauma repair [30], and it is associated with plant growth and stress resistance. Lignin can form a natural barrier to prevent external damage, which allows plants to resist diseases by enhancing the lignification of cell walls [31]. Lignin is mainly composed of three monomers: G, S, and H lignin monomers [32]. Currently, only G and S lignin monomers have been reported to be involved in disease resistance [33]. However, through confocal Raman microscopy and lignin autofluorescence detection, we found that with Psa infection, the total lignin signal intensity in kiwifruit stem cells decreased, the cells gradually collapsed, and the lignin autofluorescence intensity in xylem weakened. We speculated that the first barrier of cell-wall destruction by Psa might be due to the destruction of lignin biosynthesis. However, the specific destruction of lignin monomers needs to be further verified by high-performance liquid chromatography quantitative analysis.

The systematic analysis of the laccase gene family is valuable in understanding the effects of its structure and function on plant growth and disease resistance and for studying its overall role in disease resistance. The publication of the full kiwifruit genome provides resources for the identification of kiwifruit laccase family genes and new disease-resistance genes. To analyze the function of kiwifruit laccase genes, bioinformatics was used to determine a series of physical and chemical properties of the gene sequences. Overall, we found 55 laccase genes in the kiwifruit genome. The physical and chemical properties of genes determine their function. The physicochemical properties of kiwifruit laccases showed a predominance of hydrophilic amino acids and a mainly extracellular subcellular location (i.e., they were secreted proteins), consistent with the characteristics of laccases in citrus [34]. Gene duplication is the main driving force of laccase gene evolution in kiwifruit. The analysis of gene selection pressure showed that kiwifruit laccase genes were biased toward a positive selection effect, as is the case with the laccase gene family in eggplant [35]. Collinearity analysis of laccase genes between kiwifruit and other species showed that the correlation with tomato was the closest. Further, *cis*-element analysis showed that MeJA was responsive to most kiwifruit laccase members.

In recent years, researchers have discovered an increasing number of miRNA members that are involved in lignin biosynthesis. Specifically, miR397, miR408, and miR857 have been identified to target plant laccase genes [36]. Among these, the negative regulation of laccase genes by miR397 has been extensively studied. For instance, PbrmiR397a directly targets the lignin synthesis functional *PbrLACs*, reducing the lignin content and stone cell differentiation in pear fruits and ultimately enhancing the quality of pear fruits [37]. Similarly, AtmiR397b specifically targets *AtLAC2*, resulting in the induction of lignin deposition [38]. In cotton, *GhLAC4* knockout and ghr-miR397 overexpression significantly reduced lignin content, whereas ghr-miR397 silencing significantly increased basal lignin content [39]. In our study, 21 laccase genes were predicted as *cis*-miR397 targets. miR528 primarily targeted *ZmLAC3*, with in situ hybridization revealing its predominant expression in the vascular tissues of maize. When miR528 was suppressed or *ZmLAC3* was overexpressed, there was a significant increase in the lignin content of corn stems [40]. In our study, we investigated the expression of the laccase gene in kiwifruit stems following Psa infection. However, it remains unknown whether expression varies among different tissue parts of the stems and the specific spatial regulation of the laccase gene by miRNA397, as predicted by us. Future experiments, such as RNA ligase–mediated RLM-5′RACE and degradation group sequencing, will help identify the interacting miRNA397 after Psa infection [41] and the impact on lignin synthesis. It would also be worthwhile to explore whether miRNA397 regulates specific lignin monomers. RT-qPCR results showed that the relative expression of laccases in kiwifruit stems was higher than that in leaves, indicating that the stem had a stronger response to Psa infection than the leaves. In addition, at 5 days post-inoculation in stems, the *AcLAC2*, *AcLAC4*, *AcLAC17*, *AcLAC18*, *AcLAC26*, and *AcLAC42 *expression levels were significantly higher (*p* < 0.001) than those in the non-inoculated (0 d) control; this may indicate that these six genes may be related to resistance to kiwifruit bacterial canker. Future research will focus on whether the predicted miRNA397 has a regulatory relationship with these six genes, as well as the regulatory mechanism involved. Furthermore, the expression levels of *AcLAC42* and *AcLAC53* genes were found to reach their lowest point after Psa infection for 3 days in leaves. Hence, it is crucial to conduct further research to ascertain whether the highly expressed miRNA397 negatively regulates these two genes, thereby resulting in the suppression of laccase gene expression and the disruption of lignin synthesis.

## Conclusions

In this study, 55 laccase genes in the kiwifruit genome were systematically identified and divided into five groups. Their gene structures, phylogenetic relationships, 3D structures, evolutionary relationships, and *cis*-regulatory elements were analyzed. In addition, 21 laccase genes were found to be potential targets for *cis*-miRNA. Five days post-Psa inoculation, six genes in the stem were significantly induced in kiwifruit stems, which indicates their potential defensive role in resistance to Psa. Our research provides a reference for the application of the *AcLAC* genes in kiwifruit disease resistance breeding.

## Materials and methods

### Plant materials and Psa inoculation

The stems of biennial kiwifruit (*Actinidia chinensis* cv. Hongyang) were collected from the kiwifruit potting farm of Guizhou University (26.46°N, 106.66°E). Psa, a strong pathogenic strain of kiwifruit canker, was provided by our laboratory. Kiwifruit stems (10-cm long) were collected, surface disinfected with 0.6% NaClO for 20 min, rinsed with sterile water thrice, and dried. A 5-mm-wide wound was cut in each stem with a blade to the depth of the phloem. Then, 10 μL of Psa bacterial solution (OD 600 nm = 0.1, 10^8^ cfu/mL) was applied to the wound, and the same amount of sterile water was applied to control samples. In vitro leaf spraying inoculation was performed as follows. Healthy leaves were selected, surface disinfected (0.6% NaClO, 8 min), dried, and evenly sprayed with bacteria (10^8^ cfu/mL). In the control, the same amount of sterile water was sprayed instead of the bacterial solution. The stems and leaves were then placed in an artificial climate incubator (photoperiod: 16 h/8 h; day and night temperature: 18 °C/14 °C; relative humidity: 75%). The morbidity results were observed, and samples were taken at 0, 1, 3, 5, and 7 days after inoculation.

### Confocal raman microscopy

The kiwifruit stems infected with Psa for increasing time periods were cut using a sliding microtome, with a transverse section thickness of 10 μm. Raman imaging was performed using the WITec Alpha 300R system (Witec, Ulm, Germany). Samples were excited with a 532 nm wavelength laser at 10-mW power and a numerical aperture of 0.55 through a 50 × objective lens. Raman single spectra were acquired from selected cell parts on each slice, with an integration time of 60 s per spectrum [42]. To map in the x,y plane at a selected spatial resolution, the slice was moved in the x,y dimension, and the spectrum at each x,y position (with fixed z position) was recorded. The spectrum of each position was obtained by integrating for 1.5 s with one scan per pixel over a 60 × 60 μm area. This was repeated thrice, and the average spectrum was taken. The mapped image was generated through baseline correction of spectral peak height using LabSpec6 software (Horiba Jobin Yvon SAS, Palaiseau, France) for data acquisition and analysis. The experiment was conducted at a constant temperature of 25 °C.

### Confocal laser scanning microscopy

Stem tissues of kiwifruit infected with Psa were selected at incremental time points and fixed in FAA (50 mL 40% formaldehyde, 50 mL ice-cold acetic acid, and 90 mL 50% ethanol) for 3 days [43]. After dehydration using an ethanol series (50%, 70%, 90%, and 100%), the sections were wrapped in glycerin and covered with a 0.17-mm-thick cap. The sections were observed using a Leica fluorescence microscope (DM6000B; Leica Microsystems, Wetzlar, DE, USA) and a high-pressure mercury lamp. The self-fluorescence of kiwifruit stem lignin was stimulated under an excitation wavelength of 405 nm [44], with a continuous wave power of 50 mW and a laser intensity of 5%. Images were captured using a 40X objective lens (Leica Microsystems Wetzar, DE, USA).

### *AcLAC* identification and phylogenetic tree construction

The kiwifruit genome database (http://kiwifruitgenome.org/) was used to obtain CDS, gDNA, and protein sequences of the laccase gene family; this was achieved through a keyword search for laccase. NCBI (https://www.ncbi.nlm.nih.gov) Blastn and Blastp, as well as DNAMAN, were then used to identify members of the obtained kiwifruit laccase family. ProtParam (https://web.expasy.org/protparam) online software was used to analyze physicochemical properties, such as the number of amino acids, theoretical isoelectric point, and molecular weight, of the laccase family. SignalP 3.0 Server (http://www.cbs.dtu.dk/services/SignalP-3.0/) was used for signal peptide predictive analysis, and WoLF PSORT (https://wolfpsort.hgc.jp/) was used for predictive analysis of subcellular localization. The online software NCBI conserved domain search (https://www.ncbi.nlm.nih.gov/structure/cdd/wrpsb.cgi) with Pfam v31.0 was used to analyze the protein domains of candidate laccase genes. Sequences of *Arabidopsis thaliana* and kiwifruit were compared using MEGA X (version X), and the neighbor-joining method was used to construct a phylogenetic tree.

### Gene and 3D structure analyses of kiwifruit laccase

The GSDS website (http://gsds.cbi.pku.edu.cn/Gsds_ about.php) was used to perform predictive analysis of the gene structure. The protein spatial model was constructed using SWISS-MODEL (https://swissmodel.expasy.org/) provided by the online software ExPaSy for 3D structural homologous modeling, and ERRAT and WHATCHECK were used to detect the 3D structure of AcLAC protein in SAVES (http://nihserver.mbi.ucla.edu/SAVES/). The 3D structural drawings were created using PyMOL 2.5 software (PyMol Molecular Graphics System, version 1).

### Chromosomal distribution and gene replication of the *AcLACs*

Whole-genome information for *Arabidopsis* was downloaded from the TAIR10 database (http://www.arabidopsis.org/index.jsp). In addition, rice, grape, and tomato genomic data were downloaded from the Ensembl database (http://plants.ensembl.org/index.html). Using MCScanX software [45], the replication event of laccase gene family members was analyzed under default parameters. Combined with the chromosome position information of family members, a collinear plot was created using Circos software [46]. Tbtools [47] was used to calculate the Ka/Ks value for each replicated laccase gene, where Ka/Ks < 1 indicates purifying selection, Ka/Ks = 1 indicates neutral selection, and Ka/Ks > 1 indicates positive selection [48].

### *Cis*-regulatory element analysis and regulated miRNA prediction of kiwifruit laccase

The 2000-bp upstream nucleic acid sequence of laccase genes were extracted from the whole genome of kiwifruit (http://kiwifruitgenome.org/) using Tbtools software (v1.120). Plant CARE (http://bioinformatics.psb.ugent.be/webtools/plantcare/html/) was used for promoter *cis*-element analysis, and the *cis*-regulatory element figure was drawn using Tbtools software (v1.120). The psRNATarget server (http://plantgrn.noble.org/psRNATarget/) was used to analyze the presence of miRNA targets in the genes, and the maximum expected value was set to avoid false positives.

### RNA Extraction and qRT-PCR analysis

To identify additional kiwifruit laccase genes playing a major role in the synthesis of kiwifruit stem lignin, we analyzed the expression patterns of 18 genes that are closely related to the genes involved in Arabidopsis lignin synthesis. Kiwifruit stems from different Psa inoculation time (0, 1, 3, 5, and 7 days) were used as inoculation material 10 μL inoculation solution (OD 600 nm = 0.1, 10^8^ cfu/mL). Total RNA was extracted using a plant RNA extraction kit (GK0416; Huayueyang, Beijing, China) according to the manufacturer’s instructions and stored in the refrigerator at − 80 °C until analysis. Next, 1% agarose gel electrophoresis was used to detect RNA degradation or contamination. The NanoDrop 2000 spectrophotometer (Thermo Fisher Scientific, Waltham, MA, USA) was used to detect RNA purity and concentration. Genes were amplified with qRT-PCR primers designed using Primer Premier 5.0 software (Premier Biosoft International, San Francisco, CA, USA) and synthesized by Shanghai Shenggong Bioengineering Co., Ltd. (Shanghai, China). The primer sequences are shown in Table S4 (Supporting Information, Table S3). All samples were tested in three replicates. The 2^−ΔΔCt^ method was used to calculate the relative expression of the candidate genes.

### Data analysis

One-way analysis of variance was performed using SPSS 26.0 (IBM, Inc., Armonk, NY, USA). Charts were constructed using Origin 2022 (OriginLab, Northampton, MA, USA) and GraphPad Prism Version 8.0 (San Diego, CA, USA). Comparison of the means was carried out using Tukey’s *t*-test, and *p* < 0.05 was set as the threshold for significant differences.

### Supplementary Information

**Additional file 1: Table S1.** Basic physical and chemical characteristics and putative subcellular localization of the proteins encoded by *AcLACs*.

**Additional file 2: Supplementary Table 2.** One-to-one orthologous relationships between kiwifruit and *Solanum lycopersium, Arabidopsis thaliana,* *Vitis vinifera,Oryza sativa*.

**Additional file 3: Table S3.** List of the *AcLAC* genes with putative miR397 target sites.

**Additional file 4: Table S4.** *AcLAC *genes Real-time fluorescence quantitative analysis under Psa infection.

## Data Availability

The databases used in the study includes the whole kiwifruit genome sequence was retrieved (*A. chinensis* var. ‘Hongyang’ v3.0) from the Kiwifruit Genome Database (http://kiwifruitgenome.org/), The genome-wide protein data of rice (*Oryza sativa* L.), tomato (*Solanum lycopersicum*), and grape (*Vitis vinifera* L.) were collected from Ensembl Plants (http://plants.ensembl.org), the Arabidopsis Information Resource (TAIR) (https://www.arabidopsis.org/), iTOL (https://itol.embl.de/tree/2022041201 56,262,211,655,887,181), Expasy ProtParam (https://web.expasy.org/protparam/), Expasy Protscale (https://web.expasy.org/protscale/), PSORT Prediction (http://www.genscript.com/psort.html), MEME (https://meme-suite.org/meme/tools/meme), Conserved Domains Search database (CDD) (https://www.ncbi.nlm.nih.gov/cdd/?term =), PlantCARE (http://bioinformatics.psb.ugent.be/webtools/plantcare/html/). The psRNATarget server (http://plantgrn.noble.org/psRNATarget/). The public access to all these databases is open. The data that support the findings of this study are available from the corresponding author upon reasonable request.

## Abbreviations

Psa: *Pseudomonas syringae* pv*. actinidiae*
Laccase: LAC
kDa: Kilo Dalton
CDS: Coding sequence
3D: Three-dimensional
MeJA: Methyl jasmonate
ABA: Abscisic acid
SA: Salicylic acid
GA: Gibberellin
IAA: Auxin
qRT-PCR: Quantitative real-time polymerase chain reaction
